# How smartwatch use drives user reciprocity: The mediating effects of self-expansion and self-extension

**DOI:** 10.3389/fpsyg.2022.1041527

**Published:** 2022-11-15

**Authors:** Rong Liu, Jiawei Yang, Junwen Yao

**Affiliations:** School of Economics and Management, Nanchang University, Nanchang, China

**Keywords:** smartwatch use, self-expansion, self-extension, user reciprocity, service-dominant logic

## Abstract

People are increasingly using smartwatches in their daily lives. Scholars have focused on the drivers of the initial and continued use of smartwatches, while few studies have dealt with the outcomes of smartwatch use. Therefore, this study explores the impact of smartwatch use on user experience (self-expansion and self-extension) and user reciprocity (user loyalty and user influence) based on service-dominant logic. Data were collected through a questionnaire survey of 343 smartwatch users in China. Structural equation modeling and the bootstrapping method were applied to test the theoretical hypotheses. The results show that smartwatch use positively affects self-expansion and self-extension, both self-expansion and self-extension positively affect user loyalty and user influence, and smartwatch use affects user loyalty and user influence through self-expansion and self-extension. This research deepens our understanding of the outcomes of smartwatch use, and provides insights for smartwatch manufacturers to create more value from user reciprocity.

## Introduction

The wearable technology market has experienced steady growth in recent years and is expected to reach USD118.16 billion by 2028 (Grand View Research, 2021). Wearable technologies are generally seen as personal computing devices that can be worn by users and connected to the Internet, including smartwatches, smart glasses, and smart clothing (Basha et al., 2022). Smartwatches, in particular, have been widely commercialized and are considered to be one of the most popular wearable technologies (Bolen, 2020a). People can use smartwatches to garner various benefits, such as receiving and responding to notifications, playing music, monitoring users’ health, and making mobile payments. These benefits cover many scenarios in people’s daily lives (e.g., work, entertainment, sports, and shopping; Bolen, 2020b).

As people have begun to notice and accept smartwatches, scholars have carried out a great deal of research on smartwatches. When smartwatches first appeared on the market, the initial use of smartwatches was the main research topic (Wu et al., 2016). Studies have revealed that the initial use of smartwatches can be affected by perceived value (Choi and Kim, 2016; Hsiao and Chen, 2018), and user attitudes (Wu et al., 2016). When smartwatches moved beyond the initial adoption stage, researchers shifted their focus to the continued use of smartwatches (Dehghani et al., 2018). For example, Nascimento et al. (2018) extended the expectation confirmation model and stated that habit is the most important factor influencing the continued use of smartwatches, while Chuah (2019) found that perceived benefits and previous lifestyle incongruence influence users’ intention to continue using smartwatches through inspiration and well-being. Moreover, Shin and Biocca (2018) expanded the understanding of smartwatch users and found that upgrading behavior is not influenced by perceived usefulness, but is related to identity formation.

Although smartwatch use have become a common phenomenon among people and is considered to have major effects on people’s lives (Ogbanufe and Gerhart, 2018), little is known about the impact of smartwatch use on smartwatch users or manufacturers. To fill this research gap, this study focuses on the outcomes of smartwatch use based on service-dominant logic (S-D logic hereafter). Specifically, we argue that smartwatch use may affect user experience (self-expansion and self-extension), which further motivates smartwatch users to create value for smartwatch manufacturers through two forms of reciprocity (user loyalty and user influence).

According to S-D logic, value is not embedded in the tangible products provided by a manufacturer (i.e., value in exchange), but is co-created by the user and the manufacturer in the process of using the product (i.e., value in use; Vargo and Lusch, 2004). In this process, the users integrate their own resources with those of the manufacturers to gain benefits and experience, thus co-creating value with the manufacturers (Baumann et al., 2017). Value co-creation occurs over time and needs to be built on long-term and deep interactions (Shulga et al., 2021). Given this, when a smartwatch is used frequently and favorably, the user and the manufacturer are both likely to realize value co-creation.

Based on S-D logic, value is determined by the experience that users get while using a product (Vargo and Lusch, 2008). Self-expansion and self-extension are two key forms of experience that users can acquire when using smart products in their daily lives (Hoffman and Novak, 2018). Self-expansion refers to a person’s perception of increased self-awareness after he or she acquires new resources, knowledge, or identities from interaction with another party (Mao et al., 2019). Smartwatch use is essentially interaction between a user and a smartwatch through which the user can enhance their abilities and perceive self-expansion. Furthermore, if an object plays a role in constructing people’s self-identity, people can see the object as part of the self, thus achieving self-extension (Park and Kaye, 2019). Specifically, people who use smartwatches are likely to enrich their self-identity and experience self-extension. Thus, users can derive experiential value from smartwatch use in the form of self-expansion and self-extension.

S-D logic highlights that value co-creation is reciprocal, and a user who derives value from consuming a manufacturer’s product may provide inputs that directly or indirectly benefit the manufacturer (Vargo, 2009). Accordingly, people who get favorable experiences from using smartwatches are likely to contribute value to manufacturers in return. Thus, smartwatch users that get extraordinary experiences may make repeat purchases and exhibit user loyalty, providing direct value to manufacturers (Cetin, 2020). In addition, people may share their positive experiences with others online and create user influence, providing indirect value to manufacturers (Kumar et al., 2010).

Therefore, this study considers smartwatch use as an important activity of value co-creation and explores the impact of smartwatch use on user experience (self-expansion and self-extension) and user reciprocity (user loyalty and user influence). Specifically, we hypothesized the positive effects of smartwatch use on self-expansion and self-extension, the positive effects of self-expansion and self-extension on user loyalty and user influence, and the mediating effects of self-expansion and self-extension. We collected data of 343 smartwatch users in China through an online survey, and adopted structural equation modeling and the bootstrapping method to test the theoretical hypotheses.

The research findings contribute to the literature on smartwatches in three ways. First, unlike the literature focusing on the antecedents of smartwatch use, this study focuses on the effects of smartwatch use on users and manufacturers, expanding the streams of research into smartwatches. Second, studies have focused on the technology- and fashion-related perceptions surrounding smartwatches, such as perceived interactivity (Basha et al., 2022) and visual aesthetics (Cho et al., 2019). However, this study explores self-perceptions generated by smartwatch use. Finally, while the literature has only focused on user- and product-related variables, this study investigates variables related to manufacturers and other users beyond the focal users and products, such as user loyalty to the manufacturer and user influence on other users.

## Literature review

### Smartwatch use

Initial and continued use of smartwatches has been the focus of researchers. Many studies have shown that the initial use of smartwatches could be affected by the benefits or values provided by smartwatches and the attitude of users. In terms of the benefits or values provided by smartwatches, social value (Wu et al., 2016), perceived self-expressiveness (Choi and Kim, 2016), emotional value (Hsiao and Chen, 2018) all positively enhance users’ intention to adopt smartwatches. In addition, users’ attitudes toward using smartwatches were consistently found to promote initial use of smartwatches (Choi and Kim, 2016; Wu et al., 2016; Hsiao and Chen, 2018; Krey et al., 2019). Bolen (2020a) found that users would compare smartwatches to traditional wristwatch. In this scenario, relative advantages and financial switching costs influence users to switch from traditional wristwatch to smartwatches.

Similar to initial use, continued use of smartwatches is influenced by smartwatch-related and user-related factors. In terms of smartwatch-related factors, perceived benefits (Chuah, 2019), perceived usefulness (Nascimento et al., 2018) and aesthetic appeal (Dehghani et al., 2018) all contribute to the continued use of smartwatches. In addition, the continued use of smartwatches is also related to the perceptions and traits of users. For example, Nascimento et al. (2018) found that habit is the most critical factor in explaining the continued use of smartwatches. User satisfaction is also an important antecedent that drives users to continue using smartwatches (Ogbanufe and Gerhart, 2018). In addition, Hong et al. (2017) revealed the relationship between user innovativeness and continued use of smartwatches, and argued that user innovativeness affects continued use of smartwatches through hedonic value and utilitarian value.

It can be seen that the existing studies have advanced our understanding of the antecedents of initial and continued use of smartwatches. However, knowledge about the outcomes of smartwatch use is scarce. According to the service-dominant logic, product use is essentially a process of value co-creation. For researchers and managers, it is necessary to explore the value that smartwatch use brings to users and manufacturers.

### Service-dominant logic

Vargo and Lusch first proposed the S-D logic in Journal of Marketing in 2004 to replace the traditional goods-dominant (G-D) logic. The S-D logic asserts that service is a process in which an actor uses their own resources (knowledge and skills) to benefit another actor (Vargo and Lusch, 2008). In light with S-D logic, customers become co-creators of value and important sources for firms to gain competitive advantage. Therefore, firms no longer simply regard customers as marketing objects, but as an operant resource. Customers have made unprecedented contributions to firms’ marketing processes; firms no longer simply manufacture products or services, but help customers gain experience in the process of value creation; firms no longer simply focus on the specific value delivery, but emphasize the creation and refinement of value proposition; firms no longer simply produce and sell products, but create value together with customers. In addition, value co-creation is the creation of customer experience jointly by firms and customers (Vargo and Lusch, 2004). Customers can even gain their own experience in consuming products and services. It is now critical for firms to deliver favorable experience along the customer journey (Lemon and Verhoef, 2016). Therefore, the viewpoints of S-D logic support the influence chain of “product use—user experience—value creation,” which provides the rationale for our research model.

## Research model and hypothesis development

### Smartwatch use and user experience

Self-expansion theory contains two core components: motivation for self-expansion, which is an intrinsic motivation, and the inclusion of others in the self, which is a cognitive process (Aron and Aron, 1996). Self-expansion motivation is a core human motivation that drives people to acquire new resources, ideas, and identities to enhance their self-efficacy for achieving desired goals (Aron et al., 2006). Self-expansion is the individual perception of enhanced self-awareness after one party acquires new resources, ideas, or identities from another party (Mao et al., 2019). Studies have shown that customers are highly likely to realize self-expansion in the process of interacting with specific brands, thus leading to positive customer behaviors (Gorlier and Michel, 2020). Therefore, we argue that users will likewise achieve self-expansion in their interaction with smartwatches. First, as an innovative product, smartwatches have many novel features. Therefore, when users interact with their smartwatches, they can achieve self-expansion by acquiring new resources, ideas, and identities (Carpenter and Spottswood, 2013). Second, participating in creative activities is one of the effective ways for people to gain self-expansion (Hoffner et al., 2016). Using smartwatches itself is a highly novel and creative activity through which users can also gain self-expansion. We hypothesize the following:

*H1*: Smartwatch use positively affects self-expansion.

Self-extension refers to the subjective feeling that a person considers an object as part of the self (Belk, 1988; Ferraro et al., 2011). Self-extension theory suggests that if an object plays a role in the construction of a person’s self-identity, that person can see that object, such as property (Roster et al., 2016), as being part of the self, thus achieving self-extension (Park and Kaye, 2019). According to self-extension theory, we believe that users may realize self-extension in using smartwatches. First, the more a person uses a smartwatch, the greater the degree to which the user controls or masters the smartwatch. Mastery or control of an object is one of the ways in which people achieve self-extension (Belk, 1988), so using smartwatches may help them achieve self-extension. Second, the more a person uses a smartwatch, the more comprehensively and deeply the user knows the smartwatch. Knowing an object is another way for people to achieve self-extension (Belk, 1988), so using smartwatches may help them achieve self-extension. We hypothesize the following:

*H2*: Smartwatch use positively affects self-extension.

### Self-expansion and user reciprocity

Self-expansion plays an important role in building and maintaining relationships (Harasymchuk et al., 2021). In an ideal relationship, people may gain new resources and knowledge, thus achieving self-expansion and benefiting from intimacy (Harasymchuk et al., 2020). They tend to maintain such relationships to gain continuous self-expansion (Mattingly et al., 2019). In addition, self-expansion makes people hold positive feelings toward others with whom they have a satisfying relationship, enhancing the quality of the relationship (De Kerviler and Rodriguez, 2019). For high-quality relationships, people are willing to maintain and sustain them (Han et al., 2021). Thus, after achieving self-expansion through smartwatch use, users would maintain their connection with the smartwatch and show user loyalty.

In addition, user self-expansion may affect user influence. First, after achieving self-expansion through using a product, a person develops positive feelings toward the product (De Kerviler and Rodriguez, 2019). This enhances the quality of the user’s relationship with the product (Gordon and Luo, 2011), making the user more likely to talk about the product on the Internet and influence the activities of other users. Second, self-expansion may improve people’s self-efficacy (Dys-Steenbergen et al., 2016). Therefore, after achieving self-expansion, smartwatch users would become more confident in their product knowledge of smartwatches and more willing to share information about smartwatches online. In addition, self-expansion would enhance people’s ability to complete tasks (Mao et al., 2019). After achieving self-expansion, smartwatch users are capable of sharing information about the smartwatch online. In summary, self-expansion may encourage users to share their smartwatches online, generating user influence. Therefore, we hypothesize the following:

*H3*: Self-expansion positively affects user loyalty (H3a) and user influence (H3b).

According to S-D logic, using a product is a process of value co-creation (Vargo and Lusch, 2004). Moreover, value co-creation is inherently reciprocal (Vargo, 2009). All parties involved in value co-creation are beneficiaries and are able to obtain their desired value from value co-creation (Baumann et al., 2017). Using smartwatches could allow users to gain new resources, knowledge, and perspectives, and get value in the form of self-expansion. After reaping this value, users in turn create value for the manufacturer based on the principle of reciprocity in value co-creation. This user reciprocity can also be demonstrated through user loyalty and user influence. As such, it is hypothesized that:

*H4*: Smartwatch use positively affects user loyalty (H4a) and user influence (H4b) through self-expansion.

### Self-extension and user reciprocity

In the customer-brand relationship, customer self-extension usually results in positive outcomes for the brand, such as brand attachment (Rabbanee et al., 2020) and brand loyalty (Sprott et al., 2009). It is inferred that self-extension of smartwatch users also affects aspects of user reciprocity such as user loyalty and user influence. Seeking self-extension is a common means by which people come to know themselves (Ross and Bayer, 2021). Achieving self-extension enriches the self-concept, while losing self-extension undermines it (Israeli, 2022). To keep the integrity and continuity of their self-concept, people tend to maintain a sense of self-extension and stay connected to the object that is an extension of the self (Giordano et al., 2020). Thus, after achieving self-extension, users tend to maintain their relationship with the product and show user loyalty.

A person’s self-extension can be enhanced by unique experiences that are concrete and public (Hornik and Diesendruck, 2017). Therefore, to achieve self-extension, people tend to concretize their experiences by recording them with the help of the Internet. At the same time, they can share their experiences with others through the Internet and make their experiences public (Belk, 2013). In the context of smartwatches, users may concretize and publicize their own experience of using smartwatches and engage in user influence to maintain a sense of self-extension. We hypothesize the following:

*H5*: Self-extension positively affects user loyalty (H5a) and user influence (H5b).

According to S-D logic, the potential value of a product can be transformed into real value only when people use the product (Vargo and Lusch, 2004). Furthermore, value co-creation is inherently mutual and reciprocal (Vargo, 2009). All parties involved in the process of value co-creation will receive value (Baumann et al., 2017). People who use smartwatches come to see their smartwatches as part of themselves and experience value in the form of self-extension. After reaping this value, the users create value for the manufacturer in return based on the principle of reciprocity in value co-creation. This reciprocity of smartwatch users is reflected as their loyalty to the manufacturer and influence on other users. Therefore, smartwatch use affects self-extension, which further affects user reciprocity. We hypothesize the following:

*H6*: Smartwatch use positively affects user loyalty (H6a) and user influence (H6b) through self-extension.

**Figure 1:**
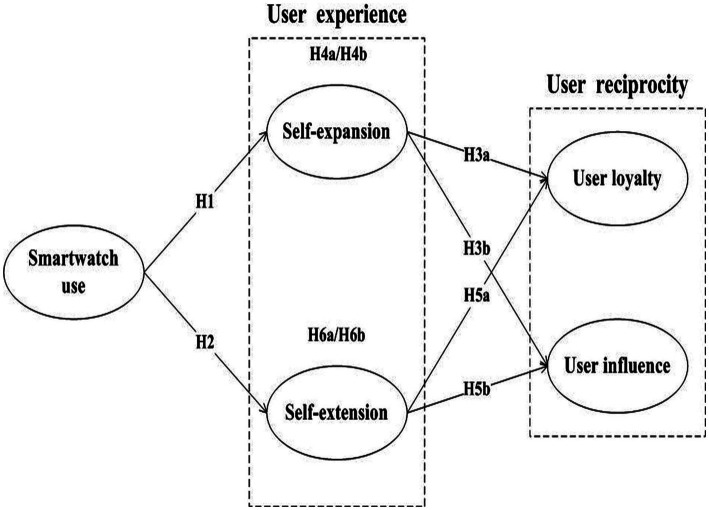
Research model.

Based on the above discussion, we construct the following research model (Figure 1).

## Materials and methods

### Questionnaire

Before designing the formal questionnaire, two tasks were completed. First, researchers used smartwatches personally to get familiar with smartwatches, such as functions of smartwatches. Second, we reviewed the relevant literature and drew on the research design of existing literature.

The questionnaire in our study is divided into a warm-up section, a main section, and a personal basic information section. In warm-up section, we firstly ask the respondents whether they have used a smartwatch. Then, we encourage them to recall the situation they used the smartwatch to further awaken their memories about smartwatch use. The main section includes the measurement of five key variables: smartwatch use, self-expansion, self-extension, user loyalty, and user influence. The personal basic information section contains gender, age, education level, and personal monthly budget.

### Measurement

The measurement items for the five core variables in this study all derived from well-established scales in existing studies, with appropriate modifications. Smartwatch use was measured by three items adapted from Ram and Jung (1991). The scale of self-expansion included three items following suggestions of De Kerviler and Rodriguez (2019) and Lee et al. (2019). Self-extension was assessed using four items adapted from Roster et al. (2016). User loyalty was measured using three items based on studies of Wolter et al. (2017) and Ramaseshan et al. (2017). User influence was assessed using three items adapted from Kumar and Pansari (2016). Smartwatch use, self-expansion, self-extension, user loyalty, and user influence were all measured using a 5-point Likert scale, with 1 being strongly disagree and 5 being strongly agree. In addition, some demographic variables, such as user gender, age, education level, and personal monthly budget, were measured with 1 item.

### Data collection

The respondents of our questionnaires are smartwatch users in China, and we surveyed them through an anonymous online survey. We collected data following two steps. First, we used “Wenjuanxing” platform to design online questionnaires and generated QR codes and web links for users to respond. Second, we posted questionnaires to online communities of smartwatch users and social networking sites to invite users to participate in the survey. To encourage smartwatch users to engage in the survey, we offered five CNY as an incentive to the respondents who finished the questionnaire.

To ensure that respondents are real smartwatch users, we set the first question as “Have you ever used smartwatches?” The respondents who replied “No” would skip the subsequent items and submit the questionnaires. As a result, 551 questionnaires were collected. Two hundred and eight invalid questionnaires were excluded following two criteria: (1) the respondents who had not used smartwatches; (2) they did not answer the questionnaire attentively. In the end, the valid questionnaires were 343. Demographic characteristics about the 343 respondents are shown in Table 1.

**Table 1:** Demographic characteristics of effective samples (*N* = 343).

Category	Range	Number	Percentage (%)
Gender	Male	154	44.9
	Female	189	55.1
Age	18–25	103	30.0
	26–30	132	38.5
	31–40	97	28.3
	41–50	8	2.3
	51 and above	3	0.9
Education level	Elementary school	2	0.6
	Middle school	5	1.5
	High school	48	14.0
	Bachelor degree	256	74.6
	Graduate degree	32	9.3
Personal monthly budget	1,500 CNY and below	16	4.7
	1,501–2000 CNY	114	33.2
	2001–2,500 CNY	71	20.7
	2,501–3,000 CNY	82	23.9
	3,001 CNY and above	60	17.5

Personal monthly budget refers to the amount of spending in daily life each month (except for housing costs, which vary largely in different cities), including food, transportation, clothing, etc.

## Results

### Common method bias

Common method bias is a possible error due to the same data source and measurement approach, which may reduce the credibility of the data and even affect the model test (Podsakoff et al., 2003). So, this study controlled common method bias in the data through procedural and statistical remedies.

Procedural controls are the ways to eliminate or minimize common method bias in the research design, such as informing the respondents that the questionnaire is anonymous and that there are no right or wrong answers and making the questions clear, specific, and easy to understand (Podsakoff et al., 2012). We applied these procedural controls to the questionnaire design. In addition, this study also used two statistical controls. Technique 1 is Harman’s one-factor test, which performs an exploratory factor analysis of all variables’ items. The results showed that the variance explained by the first factor was 48.056%, which is under the critical value of 50%, indicating that the common method bias of the data is acceptable (Fuller et al., 2016). Technique 2 is checking the correlation coefficients between the variables. If the correlation coefficient is higher than 0.9, there is common method bias in the present study (Pavlou et al., 2007). The results of the analysis showed that the correlation coefficients between the variables all ranged from 0.486 to 0.688, which were below 0.9, revealing that the common method bias of the data was not significant. In conclusion, common method bias in our data is acceptable and the data is suitable for hypothesis testing.

To test whether there is multicollinearity in our data, the variance inflation factor (VIF) was estimated. The results showed that the VIF values ranged from 1.758 to 2.270, which were less than the threshold of 3.33, indicating that there was no obvious multicollinearity among the variables (Fan et al., 2022).

### Reliability and validity test

The reliability test consists of internal consistency and composite reliability. As shown in Table 2, the Cronbach’s α values of each variable range between 0.734 and 0.827, which are all above the recommended level of 0.7, indicating that the internal consistency of each variable is high. The composite reliability of each variable ranges between 0.735 and 0.827, which is above the critical value of 0.7, which indicates each variable has adequate composite reliability.

**Table 2:** Test results for reliability and convergent validity.

Items	Factor loading
Smartwatch use Cronbach’s *α* = 0.756, CR = 0.757, AVE = 0.510	
After purchasing the smartwatch, I have used it frequently	0.690
After purchasing the smartwatch, I have used its many functions	0.709
After purchasing the smartwatch, I have used it in many situations (e.g., monitoring users’ health and making mobile payment)	0.742
Self-expansion Cronbach’s *α* = 0.784, CR = 0.784, AVE = 0.548	
The smartwatch has enhanced my ability to accomplish things	0.748
The smartwatch has increased my knowledge	0.736
The smartwatch has made me a better person	0.737
Self-extension Cronbach’s *α* = 0.734, CR = 0.735, AVE = 0.481	
I have felt a personal connection between the smartwatch and me	0.690
I have considered the smartwatch to be a part of myself	0.719
The smartwatch have been an important indication of who I am	0.671
User loyalty Cronbach’s *α* = 0.822, CR = 0.822, AVE = 0.606	
I will be loyal to the manufacturer’s smartwatch	0.785
I will purchase the manufacturer’s smartwatch again	0.792
The manufacturer’s smartwatch is my first choice for the future	0.758
User influence Cronbach’s *α* = 0.827, CR = 0.827, AVE = 0.615	
I will talk about the experience of using the smartwatch online	0.777
I will discuss the benefits that I get from the smartwatch with others online	0.777
I will mention the smartwatch in my conversations online	0.799
Model fit index: *χ*^2^(80) = 140.596, *χ*^2^/*df* = 1.757 SRMR = 0.029, RMSEA = 0.047, CFI = 0.975, TLI = 0.967	

The validity of the variables can be assessed by convergent validity and discriminant validity. This study conducted a confirmatory factor analysis to test the convergent validity of all variables by employing Mplus 8.3. As shown in Table 2, the factor loadings of all items range from 0.671 to 0.799, most of them are above the cut-off value of 0.7. The average extracted variance (AVE) of all variables (except self-extension) is greater than 0.5, exceeding the recommended threshold. The results reveal that the measurement model fits well with the data (*χ*^2^/*df* = 1.757; SRMR = 0.029; RMSEA = 0.047; CFI = 0.975; TLI = 0.967). Thus, the scale of this study has strong convergent validity. The discriminant validity can be assessed by comparing the relationship between the square root of AVE and the correlation coefficients. As shown in Table 3, the square root of AVE of each variable is higher than the correlation coefficient of the variable with other variables, and the discriminant validity of the scale is good. In conclusion, the scale used in this study has adequate reliability and validity.

**Table 3:** Test results for discriminant validity.

Variables	Smartwatch use	Self-expansion	Self-extension	User loyalty	User influence
Smartwatch use	0.714				
Self-expansion	0.486^**^	0.740			
Self-extension	0.650^**^	0.639^**^	0.694		
User loyalty	0.587^**^	0.606^**^	0.688^**^	0.778	
User influence	0.510^**^	0.579^**^	0.651^**^	0.645^**^	0.784
Mean	3.9815	3.7036	3.7444	3.7133	3.7765
Standard deviation	0.6504	0.7668	0.7172	0.8274	0.7765

^**^Suggests *p* < 0.01. The diagonal elements are the square root of average variance extracted (AVE). Off-diagonal elements are the correlations among variables.

### The main effects

This study applied Mplus 8.3 to test the main effects, and the results are shown in Table 4. In the model, we regressed smartwatch use on self-extension and self-extension, self-expansion on user loyalty and user influence, and self-extension on user loyalty and user influence, respectively. As shown in Table 4, the regression coefficients of smartwatch use on self-expansion and self-extension are 1.036 (*p* < 0.001) and 1.259 (*p* < 0.001), respectively, indicating that smartwatch use positively affects self-expansion and self-extension, and H1 and H2 are supported. The regression coefficients of self-expansion on user loyalty and user influence are 0.297 (*p* < 0.05) and 0.296 (*p* < 0.05), respectively, revealing that self-expansion would generate user loyalty and user influence, and H3a and H3b are supported. The regression coefficients of self-extension on user loyalty and user influence are 0.746 (*p* < 0.001) and 0.615 (*p* < 0.001), respectively, illustrating that the deeper the degree of self-extension, the more likely user loyalty and user influence appear, and H5a and H5b are supported.

**Table 4:** Analysis results for the main effects.

Hypothesis	Estimate	Standard error	*t*-value	Results
H1	1.036	0.141	7.353^***^	Supported
H2	1.259	0.122	10.300^***^	Supported
H3a	0.297	0.142	2.090^*^	Supported
H3b	0.296	0.118	2.517^*^	Supported
H5a	0.746	0.140	5.329^***^	Supported
H5b	0.615	0.129	4.773^***^	Supported

^*^Suggests *p* < 0.05 and ^***^suggests *p* < 0.001.

### The mediating effects

This study employed Mplus 8.3 and used the bootstrapping method to test mediating effects of self-expansion and self-extension, and the results are shown in Table 5. In the model, we treated smartwatch use as the independent variable, user loyalty and user influence as the dependent variables, and self-expansion and self-extension as the mediating variables. Compared with the traditional three-step mediation test of Baron and Kenny (1986) and the Sobel mediation test, the bootstrapping method can be used to solve a wide range of inference problems and is particularly suitable in the absence of *a priori* information on the statistics (Zhao et al., 2010). For example, the bootstrapping method does not require the data to be normally distributed (Shin et al., 2022). All bootstrap analyses were performed using 5,000 replicate samples to generate bias-corrected 95% confidence intervals. If the confidence interval excludes 0, the corresponding effect is significant (Zhao et al., 2010).

**Table 5:** Analysis results for the mediation effects.

Hypothesis	Estimate	Standard error	LLCI	ULCI	Results
H4a	0.308	0.153	0.046	0.650	Supported
H4b	0.307	0.126	0.106	0.595	Supported
H6a	0.940	0.204	0.611	1.407	Supported
H6b	0.775	0.179	0.477	1.160	Supported

From Table 5, the mediating effect of smartwatch use on user loyalty through self-expansion is 0.308 and the confidence interval excludes 0. The mediating effect of smartwatch use on user influence through self-expansion is 0.307 with a confidence interval excluding 0. The mediating effect of smartwatch use on user loyalty and user influence through self-extension is 0.940 and 0.775, respectively. And, their confidence intervals all exclude 0. None of the confidence intervals for the mediating effects includes 0. Therefore, the mediating effects are all significant and H4a, H4b, H6a, and H6b are supported.

Based on the above results, it can be seen that smartwatch use affects user loyalty and user influence through self-expansion. In addition, self-extension also mediates the relationship between smartwatch use, user loyalty and user reciprocity. These findings reveal the roles of self-expansion and self-extension of smartwatch users in value co-creation, which is significant for scholars and managers to understand the experiences and behaviors of smartwatch users.

## Discussion

This study empirically examines the effects of smartwatch use on user experience (self-expansion and self-extension), and the effects of self-expansion and self-extension on user reciprocity (user loyalty and user influence). In addition, this study also regarded self-expansion and self-extension as mediating variables and inferred that smartwatch use would affect user reciprocity through self-expansion and self-extension. The results of the study are as follows.

First, smartwatch use positively affects self-expansion and self-extension. By using smartwatches, users can gain new knowledge, ability and identities and realize self-expansion. Meanwhile, they will see smartwatches as part of their self-awareness and experience self-extension. Therefore, the more a person uses a smartwatch, the more likely they are to experience self-expansion and self-extension. These findings provide empirical support for S-D logic. S-D logic argues that product use could create value for users (Vargo and Lusch, 2004) and that user experience is an important aspect to assess the value of product use (Vargo and Lusch, 2008). In our study, smartwatch use provides the experience of self-expansion and self-extension for users. Furthermore, similar to Ram and Jung’s study, these findings emphasize the importance of product use. Ram and Jung (1991) focus on product use in the context of durable goods and found product use affects consumer satisfaction through use disconfirmation.

Second, self-expansion positively affects user loyalty and user influence, and plays a mediating role in the relationship between smartwatch use and user loyalty (or user influence). When users obtain the value of self-expansion, they will show user loyalty and user influence, giving back to the related manufacturer directly or indirectly due to the reciprocity principle in value co-creation. In addition, as a value co-creation activity, smartwatch use provides users with self-expansion and motivates them to contribute value to the manufacturer in return. Therefore, smartwatch use affects user loyalty and user influence through self-expansion. This is consistent with views of S-D logic. Smartwatch use is a process of value co-creation (Baumann et al., 2017). In this process, users gain self-expansion, while manufacturers reap direct and indirect value through user loyalty and user influence, respectively. Furthermore, similar to many studies, the present study introduces self-expansion in the individual-organization relationship and reveals the positive effects of self-expansion. For example, self-expansion is considered to strengthen relationship quality and consumer-brand identification in the context of luxury brands (De Kerviler and Rodriguez, 2019). Gorlier and Michel (2020) also argue that self-expansion mediates the positive effects of reward extraordinary on brand evaluation, recommendation, and identification.

Third, self-extension is positively related to user loyalty and user influence. Moreover, self-extension mediates the effect of smartwatch use on user loyalty and user influence. After achieving self-extension, based on the principle of reciprocity in value co-creation, smartwatch users would contribute value back to smartwatch manufacturers in the forms of user loyalty and user influence. Smartwatch use is a value co-creation process, in which smartwatch users can achieve self-extension and then provide value to smartwatch manufacturers directly or indirectly. Therefore, smartwatch use has an impact on user loyalty and user influence through self-extension. These findings echo the premises of S-D logic in the context of smartwatch use. Smartwatch use is a way for users and manufacturers to achieve value co-creation (Vargo and Lusch, 2004). Besides, it is not strange that users gain self-extension in using smartwatches. On the contrary, digital technologies provide more means for people to achieve self-extension (Belk, 2013). For instance, Ross and Bayer (2021) argue that people regard their mobile phone as an extension of the self. Schweitzer et al. (2019) also draw on self-extension theory to explore the relationship between users and voice-controlled smart devices.

## Conclusion

This study focuses on the outcomes of smartwatch use and finds that smartwatch use positively affects self-expansion and self-extension, which in turn affect user reciprocity (user loyalty and user influence). Based on S-D logic, this study reveals the process by which smartwatch use creates value for users and manufacturers, enriches the relevant literature on smartwatches, and can also provide useful insights for manufacturers to manage user value.

### Theoretical contributions

First, this study enriches research on smartwatches and emphasizes the importance of smartwatch use. Existing research has mainly focused on the drivers of initial and continued use of smartwatches, neglecting the outcomes of smartwatch use. However, S-D logic emphasizes that using a product is a way to achieve value co-creation. To fill in this gap, we deal with the outcomes of smartwatch use, explores the direct impact of smartwatch use on user experience and the indirect impact on the value of manufacturers, and highlights the importance of smartwatch use.

Second, this study broadens the application context of self-expansion and self-extension theories. The literature has rarely examined the antecedents and consequences of self-expansion and self-extension in the relationship between smartwatches and users, and the context of the application of these theories is relatively limited. Thus, we innovatively utilize the theories of self-expansion and self-extension in the setting of smartwatch use, and find that self-expansion and self-extension are both important outcomes of smartwatch use and potential mechanisms for the effects of smartwatch use. These findings lead to richer contexts for the application of self-expansion and self-extension theories.

Third, this study provides empirical evidence to support S-D logic. S-D logic holds that using products is a source of value. Consistent with this view, this study finds that smartwatch use creates the experience of self-expansion and self-extension for users, which in turn drives user loyalty and user influence. Both users and manufacturers gain value from smartwatch use. In addition, S-D logic suggests that value co-creation is inherently reciprocal. This study shows that users can achieve self-expansion and self-extension by using the smartwatches provided by manufacturers. Meanwhile, in light with the principle of reciprocity in value co-creation, users also provide value to the manufacturers in the form of user loyalty and user influence.

### Practical implications

First, smartwatch manufacturers should make efforts to increase and improve people’s smartwatch use. To begin with, smartwatch manufacturers should provide users with detailed and easy-to-understand user manuals or set up a library of frequently asked questions (FAQ) on their official website, which may increase users’ understanding of smartwatches. In this case, users are more likely to use smartwatches in their daily lives. In addition, smartwatch manufacturers should strengthen the training of after-sales service agents to ensure that they can answer users’ questions about smartwatches timely. In this way, users would use smartwatches more smoothly and conveniently and thus improve the level of their smartwatch use.

Second, smartwatch manufacturers should pay attention to the management of user experience. First of all, smartwatch manufacturers should regularly upgrade the operating system of smartwatches. A high level of system quality may improve smartwatch users’ experience. A constantly updated operating system may also enhance users’ perception of the innovativeness of smartwatches. Furthermore, smartwatch manufacturers should focus on the establishment and management of user online communities and encourage users to share their experiences of using smartwatches with each other. The interaction between smartwatch users in online communities is also beneficial to the user experience. Finally, smartwatch manufacturers should enhance cooperation with other manufacturers of Internet of Things (IoT) devices, and improve the ability of smartwatches to connect with other IoT devices. This would increase the functions of smartwatches and enrich the users’ experience of using smartwatches.

### Limitations and future studies

First, this study does not explore the boundary conditions of the impact of smartwatch use on its outcomes. The impact of smartwatch use on user self-expansion and self-extension may vary for different smartwatch users. For example, it is revealed that people who are more similar with each other in a relationship are more likely to achieve self-expansion (Mao et al., 2019). Future research could investigate some potential variables, such as users’ personality traits, that moderate the effect of smartwatch use on subsequent outcomes.

Second, our research findings are only based on the survey data of smartwatch users in China. Cultural and economic backgrounds may influence people’s acceptance and use of smartwatches; therefore, the findings may not be generalizable to other countries. Future studies could be conducted in other societies to improve the generalizability of the findings.

Third, this study only focuses on the impact of a single type of wearable technology, smartwatches, on users and manufacturers. However, wearable technologies cover many other products, such as smart glasses and smart clothing, whose impact on society is still unknown. Therefore, it is necessary for other researchers to explore the impact of other devices on people to improve our understanding of wearable technologies.

## Data availability statement

The raw data supporting the conclusions of this article will be made available by the authors, without undue reservation. Requests to access these datasets should be directed to JY, y2362146000@gmail.com.

## Ethics statement

Ethical review and approval was not required for the study on human participants in accordance with the local legislation and institutional requirements. Written informed consent for participation was not required for this study in accordance with the national legislation and the institutional requirements.

## Author contributions

RL contributed to conceptualization, methodology, writing—review and editing, project administration, funding acquisition, and supervision. JiY contributed to conceptualization, methodology, and writing—review and editing. JuY performed conceptualization, methodology, writing—review and editing, funding acquisition, and supervision. All authors contributed to the article and approved the submitted version.

## Funding

This work was supported by the National Natural Science Foundation of China (72062021), the Social Science Planning Project of Jiangxi Province (19JY47), and the Research Project on Humanities and Social Sciences for Universities in Jiangxi Province (JJ19219).

## Conflict of interest

The authors declare that the research was conducted in the absence of any commercial or financial relationships that could be construed as a potential conflict of interest.

## Publisher’s note

All claims expressed in this article are solely those of the authors and do not necessarily represent those of their affiliated organizations, or those of the publisher, the editors and the reviewers. Any product that may be evaluated in this article, or claim that may be made by its manufacturer, is not guaranteed or endorsed by the publisher.
